# Occurrence of *Diopatra marocensis* (Annelida, Onuphidae) in the eastern Mediterranean

**DOI:** 10.3897/zookeys.445.8464

**Published:** 2014-10-13

**Authors:** Melih Ertan Çinar, Kristian Fauchald, Ertan Dagli

**Affiliations:** 1 Ege University, Faculty of Fisheries, Department of Hydrobiology, 35100, Bornova, Izmir, TURKEY; 2 National Museum of Natural History, Smithsonian Institution, MRC 0163 PO Box 37012, Washington DC, 20012−7012, USA

**Keywords:** Polychaeta, Levantine Sea, Aegean Sea, Turkey

## Abstract

The present study deals with the presence of *Diopatra marocensis* in the eastern Mediterranean. This species is small-sized and inhabited muddy bottom near the opening of rivers or lagoons [salinity range: 33−39‰] in the Aegean and Levantine Seas, and reached a maximum density of 90 ind.m^-2^ in Mersin Bay. This species might be an alien species that was introduced from the East Atlantic (near Gibraltar) to the eastern Mediterranean via ballast water of ships, as it has never been reported from the western Mediterranean Sea.

## Introduction

The genus *Diopatra* Audouin & Milne-Edwards, 1833 is represented by 59 species world-wide (Paxton 2014) and only by two species (*Diopatra neapolitana* Delle Chiaje, 1841 and *Diopatra micrura* Pires, Paxton, Quintino & Rodrigues, 2014) in the Mediterranean Sea (Delle Chiaje 1841; Arias and Paxton 2014). *Diopatra neapolitana* is a large species, maximally reaching a length of 39 cm in Izmir Bay (Dagli et al. 2005), 50 cm on the coast of France (Fauvel 1923) and 73 cm on the coast of Portugal (Pires et al. 2012). It occurs in vegetated and unvegetated muddy sand bottoms in the shallow-water benthic environments of the Mediterranean Sea and has ecological and economic importance in the region. It forms dense populations in organically enriched sediments (i.e. 198 ind.m^-2^ and 408 g.m^-2^ in the Aegean Sea) and has been overexploited by diggers for the fish bait trade (Dagli et al. 2005). The large and strong tube of this species, which partly protrudes from the sediment surface, acts as a sediment and substrate stabilizer that enhances local biodiversity (Thomsen and McGlathery 2005).

*Diopatra neapolitana* was previously regarded as a widely distributed species from the Atlanto-Mediterranean to the Indo-Pacific regions (see Dagli et al. 2005). After re-examinations of the specimens that were previously identified as *Diopatra neapolitana* from Japan (Okuda 1938; Choe 1960) and Singapore (Tong and Chou 1996), it was revealed that they in fact belonged to *Diopatra sugokai* Izuka, 1907 and *Diopatra claparedii* Grube, 1878, respectively (Paxton 1998), indicating its restricted distributional pattern. Since the study made by Paxton (1993), 14 new *Diopatra* species have been described in the world’s oceans, some of which co-occurred with *Diopatra neapolitana* such as *Diopatra biscayensis* Fauchald, Berke & Woodin, 2012, *Diopatra cryptornata* Fauchald, Berke & Woodin, 2012, *Diopatra marocensis* Paxton, Fadlaoui & Lechapt, 1995 and *Diopatra micrura* (Paxton et al. 1995; Fauchald et al. 2012; Pires et al. 2012)

During the biodiversity and pollution-monitoring projects performed along the coasts of Turkey between 2005 and 2012, we came across small individuals of a *Diopatra* species, especially in the eastern part of the Levantine Sea (in Iskenderun and Mersin Bays) that were first identified as *Diopatra* sp. (Çinar et al. 2012) and later as *Diopatra marocensis*. The present study re-describes this species and gives some notes on its ecological and biological features.

## Material and methods

**Figure 1.**
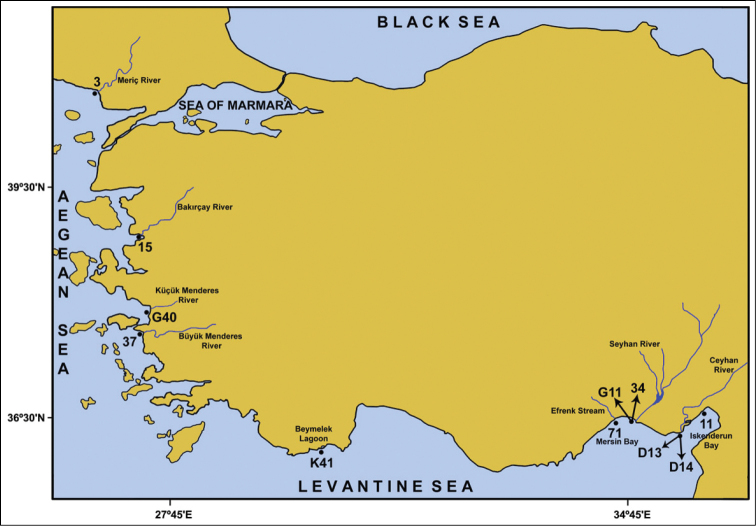
Map of the study area.

Specimens of *Diopatra marocensis* were collected at 7 stations in the Levantine Sea (stations K41, 71, G11, 34, D13, D14 and 11) and at 4 stations in the Aegean Sea (stations 3, 15, G40, 37) between 2005 and 2012, using a Van Veen grab (sampling an area of 0.1 m^-2^) and a dredge (stations D13 and D14) (Figure 1). Benthic material taken from stations was passed through 0.5 mm mesh and the retained material was fixed with 4% formaldehyde. In the laboratory, the material was washed with tap water, sorted under a stereomicroscope and preserved in 70% ethanol. The biometrical features of the *Diopatra* specimens such as the body length (excluding palps), width (excluding parapodia), H+10 (length comprising the head and the first 10 chaetigers) and the length of chaetae were measured by using an ocular micrometer. Drawings were made with the aid of a camera lucida. Specimens were deposited in the Museum of Faculty of Fisheries, Ege University (ESFM).

## Description of species

### Diopatra marocensis

Taxon classificationAnimaliaEunicidaOnuphidae

Paxton, Fadlaoui & Lechapt, 1995

**Figure 2.**
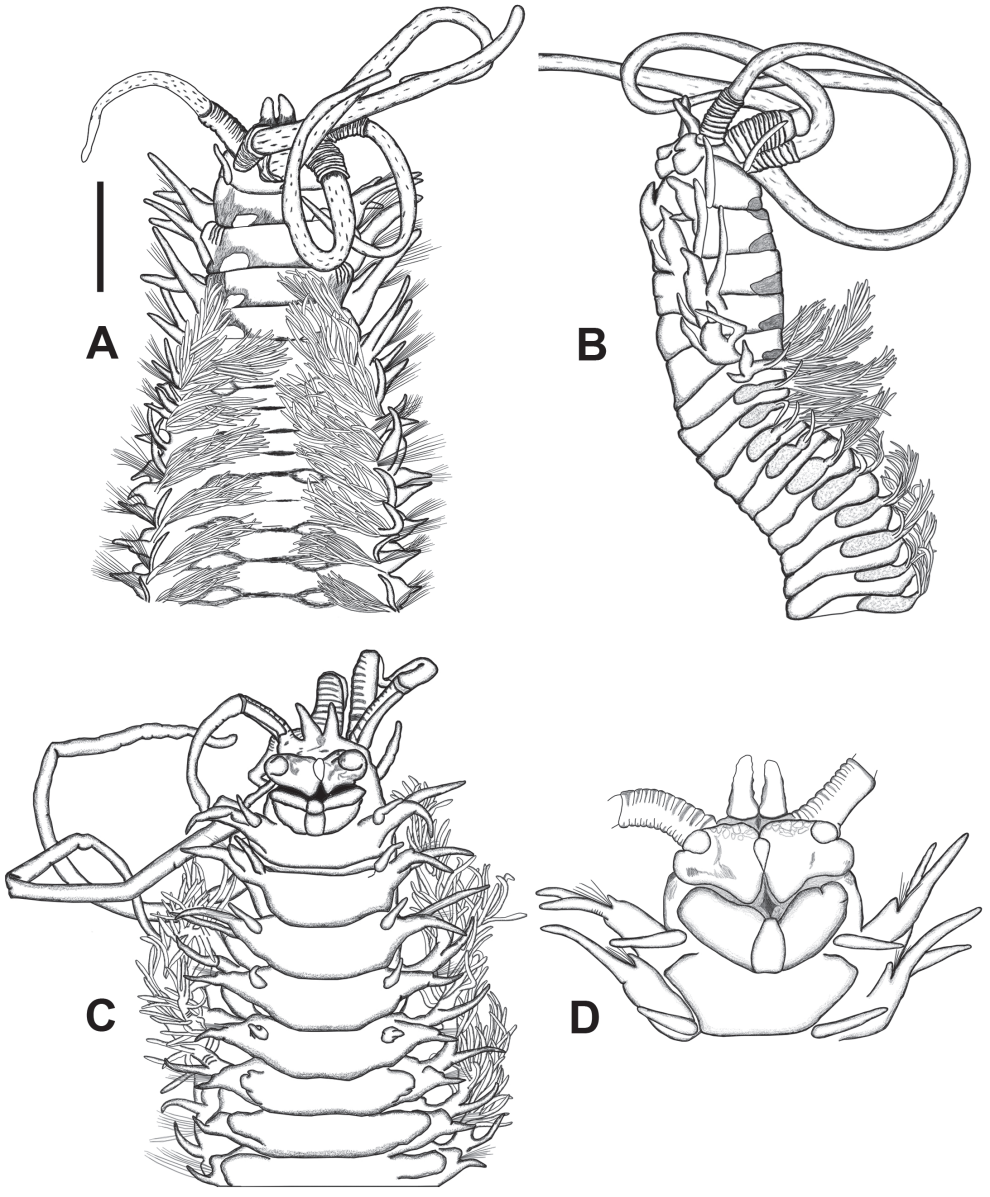
*Diopatra marocensis*. **A** Anterior part, dorsal view (ESFM−POL/2011−48) **B** Anterior part, lateral view **C** Anterior part, ventral view **D** Anterior end, ventral view. Scale bar: **A** = 2 mm, **B** = 1.80 mm, **C** = 1.88 mm, **D** = 0.73 mm.

**Figure 3.**
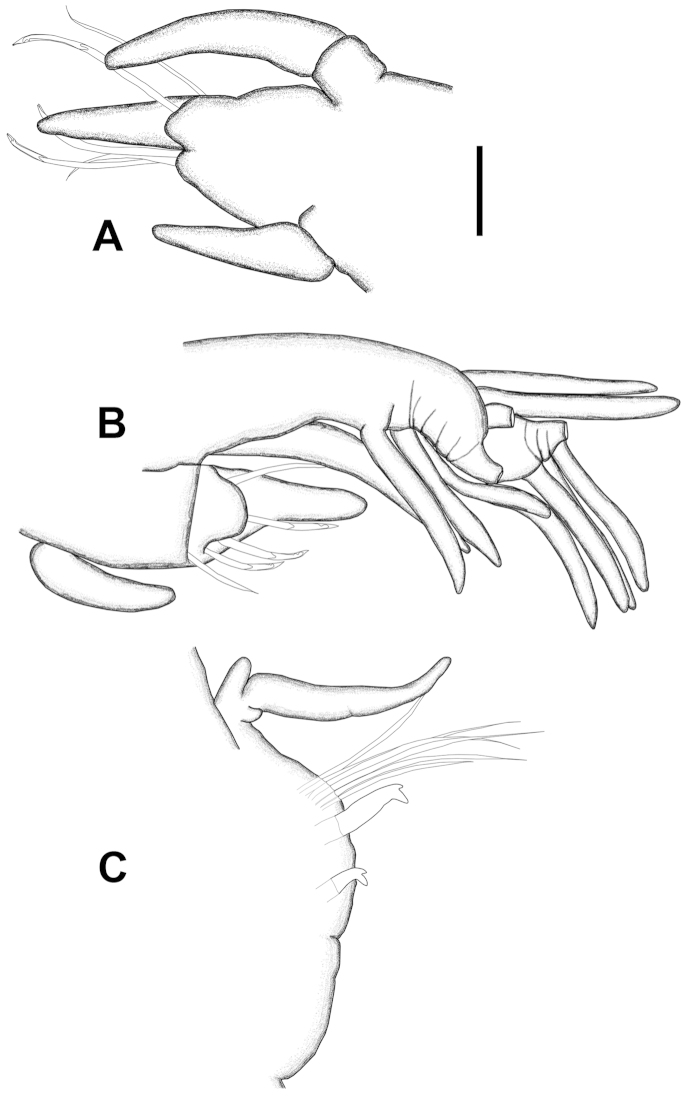
*Diopatra marocensis*. **A** Parapodium of chaetiger 1 (ESFM−POL/2009−300), anterior view **B** Parapodium of chaetiger 4, anterior view **C** Parapodium of chaetiger 43, anterior view. Scale bar: **A** = 200 µm, **B** = 96 µm, **C** = 173 µm.

**Figure 4.**
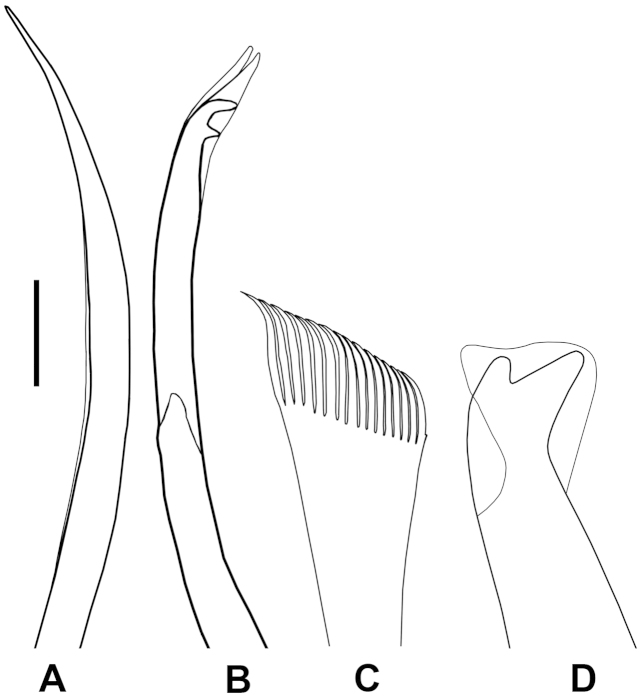
*Diopatra marocensis*. **A** Limbate chaeta, chaetiger 1 (ESFM−POL/2009−300) **B** Bidentate pseudocompound hook, chaetiger 1 **C** Pectinate chaeta, chaetiger 35 **D** Subacicular hook, chaetiger 35. Scale bar: **A** = 20 µm, **B** = 15 µm, **C** = 23 µm, **D** = 46 µm.

**Figure 5.**
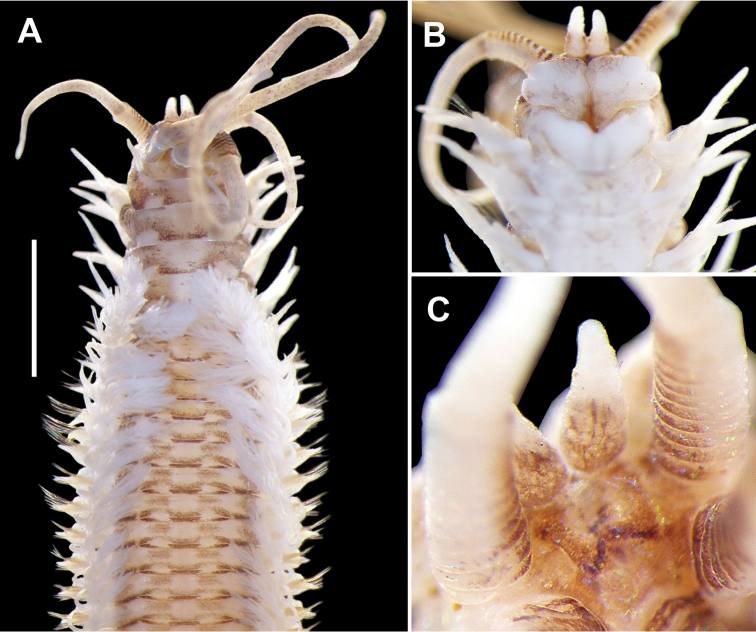
*Diopatra marocensis*. **A** Anterior part, dorsal view (ESFM−POL/2009−300) **B** Anterior end, ventral view **C** Anterior end, dorsal view. Scale bar: **A** = 2 mm, **B** = 1.18 mm, **C** = 0.18 mm.

Figures 2 , 3 , 4 , 5

Diopatra marocensis : Paxton et al. 1995: 949–955, fig. 1; Fauchald et al. 2012: 51, fig. 2a–b.

#### Material examined.

ESFM−POL/2009−196, 29.04.2009, Mersin Bay, near the opening of Seyhan River [salinity: 34‰], station 34, 36°43'33"N, 34°52'11"E, 9 m, mud; ESFM−POL/2005−1511, 1 September 2005, Kale, K41, near Beymelek Lagoon [salinity: 39‰], 36°14'29"N, 30°01'33"E, 5 m, gravelly sand, 1 specimen; ESFM−POL/2005−107, 10 September 2005, D13, near the opening of Ceyhan River [salinity: 39‰], 36°33'22"N, 35°34'17"E, 10 m, muddy sand, 8 specimens; ESFM−POL/05−295, 10 September 2005, İskenderun Bay, D14, near the opening of Ceyhan River [salinity: 38‰], 36°32'51"N, 35°34'37"E, 25 m, 3 specimens; ESFM−POL/2005−1396, 17 September 2005, Mersin Bay, G11, near the opening of Seyhan River [salinity: 38‰], 36°45'47"N, 34°51'54"E, 5 m, mud, 14 specimens; ESFM−POL/2005−2086, 8 October 2005, Kuşadası [salinity: 39‰], G40, 37°52'00"N, 27°15'27"E, 10 m, sandy mud, 3 specimens; ESFM−POL/2009−34, 4 February 2009, Mersin Bay, station 34, near the opening of Seyhan River [salinity: 38‰], 36°43'33"N, 34°52'11"E, 9 m, mud, 2 specimens; ESFM−POL/2009−300, 29 April 2009, Mersin Bay, station 34, near the opening of Seyhan River [salinity: 34‰], 36°43'33"N, 34°52'11"E, 9 m, mud, 4 specimens; ESFM−POL/2009−281, 20 October 2009, Mersin Bay, station 34, near the opening of Seyhan River [salinity: 38‰], 36°43'33"N, 34°52'11"E, 10 m, mud, 2 specimens; ESFM−POL/2011−48, 23 August 2011, Enez, near the opening of Meriç River [salinity: 33‰], station 3, 40°43'41.5"N, 26°02'03.1"E, 4 m, sandy mud, 1 specimen; ESFM−POL/2011−256, 28 August 2011, near the opening of Bakırçay River [salinity: 39‰], station 15, 38°55'11"N, 26°58'50"E, 4 m, sandy mud, 1 specimen; ESFM−POL/2011−255, 1 September 2011, near the opening of Büyük Menderes River [salinity: 39‰], station 37, 37°32'39"N, 27°10'28"E, 5 m, sandy mud, 2 specimens (juvenile); ESFM−POL/2011−254, 10 September 2011, Mersin Bay, near the opening of Seyhan River, station 71 [salinity: 37‰], 36°47'00"N, 34°38'06"E, 6 m, sandy mud, 1 specimen; ESFM−POL/2012−1, 17 June 2012, Iskenderun Bay, Yumurtalık [salinity: 39‰], station 11, 36°50'27"N, 35°54'32"E, 12 m, mud, 6 specimens.

#### Description.

All specimens incomplete, except for juvenile specimens from stations G40 and 37, and a mature specimen from station 71 (ESFM−POL/2011−254); having 27 mm body length, 1.3 mm body width (chaetiger 5) and 102 chaetigers. Large specimen incomplete, 35 mm long (H+10 = 5 mm), 2.1 mm wide, with 83 chaetigers. Body somewhat semicircular, anterio-ventral side flat in most specimens; a ventral groove in some highly contracted specimens. Anterior end including first 2 chaetigers larger than following chaetigers (Figures 2A, B, 5A). After chaetiger 15, cross section of segments becoming rectangular; ventrum of segments after chaetiger 15 with a vertical groove, extending back to posterior end. Dorsal side of prostomium, frontal lips, anterior faces of palpophores and antennophores with brown pigmentation (Figures 2A, 5A). In most specimens, a “w”-shaped brownish marking present on anterior side of prostomium (Figure 5C). Palpostyles and antennostyles with bar-shaped brownish pigments scattered on surface (Figure 2A, 5A). Body color pale brownish with a distinct dark brown color pattern on dorsal side of anterior segments (on first 20−25 chaetigers). Color pattern like eyeglasses, lying on posterior part of each segment upside down (Figure 2A, 5A). Only one specimen (ESFM−POL/2009−281) having irregular dark brown pigmentations on anterio-ventral side, other specimens without color markings on ventral side.

Antennae and palps covering dorsum of prostomium, emerging near posterior part of prostomium. One pair of pyriform, subulate or digitiform frontal lips on anterior part of prostomium; large specimen having an anomaly; with three frontal lips; on left side of prostomium, two lips attached with each other at base present. Upper lips, massive, medially distinctly separated; with a distinct, elongated papilla between lips (no ridge between halves); each lip cushion shaped, with large, bulbous distal papilla (Figure 2C, D, 5B). Mouth bordered by high ridges. Lower lips tripartite, with a triangular median section and paired high wings laterally (Figure 2D). Palps reaching posterior part of chaetiger 3. Tips of antennae missing in large specimen, reaching chaetiger 11 in other specimens. Palpophores with 10 rings; maximally 11. Antennophores with 12 rings. Palpophores and antennophores without lateral projections. One pair of small sphaerical eyes clearly discernable on juveniles, located near bases of lateral antennae (Figure 5C); due to dense pigmentation, eyes indiscernible in larger worms. Two large, crescentic nuchal organs present on posterio-dorsal sides of prostomium.

Peristomium shorter than first chaetiger, narrow on dorsal side, becoming larger laterally; ventral side as long as dorsal side. Peristomial cirri present, emerging anterio-dorsal side of peristomium; digitiform, longer than peristomium, extending to posterior part of prostomium (Figure 2A). First four or five parapodia projecting laterally; from chaetiger 6 to end of body, parapodia mainly projecting dorsally. First four or five parapodia with distinct, thick proximal part, enlarging distally; posterior ones conical, decreasing in length, becoming short cones in last chaetigers. First parapodia located dorsally, projecting laterally, second to fifth parapodia placed and directed laterally. In first three chaetigers, postchaetal lobe digitiform, extending beyond tips of dorsal cirri (Figure 3A); following chaetigers with shorter postchaetal lobe (Figure 3B, C). Prechaetal lobe of chaetigers 1−3, bilobed; dorsal part larger than ventral one. Dorsal cirri digitiform, emerging on dorsal side of parapodia; cirrophores well developed.

Ventral cirri digitiform on chaetigers 1−4, emerging from posterio-ventral side of parapodia (Figure 3A, B); tips extending just beyond prechaetal lobe; those on chaetiger 5 shorter, more or less triangular with bulbous base; those on chaetiger 6 and remaining ones as thick glandular flattened pads. Pads becoming larger on chaetiger 18−20, then gradually decreasing in size and remaining as rounded swellings in posterior parapodia (Figure 3C).

Upper fascicle of chaetiger 1 with 2 slender limbate chaetae (Figure 4A), ca. 400 µm long; lower fascicle with 4 pseudocompound hooks; distinctly bidentate, distance between pseudocompound fracture and tip of chaeta maximally 90 µm long; having long (distance between tip of chaeta and tip of hood 15 µm long), pointed hoods (Figure 4B). One aciculum penetrating within dorsal cirri and three aciculae in parapodia; tips extending beyond postchaetal lobe, resembling short simple chaetae. Parapodia 2 with 2 limbate chaetae and 5 pseudocompound hooks; resembling those on chaetiger 1. Superior fascicle of chaetiger 4 with 2 limbate chaetae, ca. 325 µm long. Inferior fascicle of chaetiger 4 with 1 limbate chaeta (225 µm long) and 3 pseudocompound hooks; distance between pseudocompound fracture and tip of chaeta maximally 65 µm long. Unmodified parapodia with serrated limbate chaeta, pectinate chaeta (first appeared on chaetiger 6) and subacicular hook (first appearing on chaetiger 13). Chaetiger 13 with 8 serrated limbate chaetae, 2 pectinate chaetae and one subacicular hook; limbate chaetae maximally 175 µm long; pectinate chaetae with oblique tips, having 16−18 thin, equal teeth (Figure 4C); subacicular hook strongly bidentate, light amber-coloured, with truncated hood, becoming double from chaetiger 14 onwards (Figure 4D).

Branchiae starting from chaetiger 4 on majority of specimens; four specimens possessing first branchiae on chaetiger 5. Branchiae with more than 10 spiraled whorls of long filaments (15 spiraled whorls on large specimen); decreasing gradually in number of whorls and in size after chaetiger 12 (7 spiraled whorls on chaetiger 15, 3 spiraled whorls on chaetiger 30); having 2 filaments after chaetiger 35, one filament after chaetiger 37, becoming a short papilla between chaetiger 40 and 46, absent posteriorly.

Mandibles partly sclerotinized (proximal parts) with calcareous distal cutting figs, rounded tip with distal indentations. Maxillae distinctly sclerotinized along cutting edges; supporting structures barely sclerotinized. Maxillary formula: Mx I = 1 + 1; Mx II = 8 + 7; Mx III = 7 + 0; Mx IV = 7 + 9; Mx V = 1 + 1.

Pygidium with two pairs of pygidial cirri; ventral pair (0.5 mm) longer than dorsal pair (0.2 mm).

Tube parchment-like, cylindrical, surface covered by debris comprising mud with shell fragments of *Abra alba* (Wood W., 1802) and *Parvicardium exiguum* (Gmelin, 1791), and plant debris drifted from rivers.

#### Ecology.

This species was found near the opening of rivers or lagoons [salinity range: 33−39‰] in the area between 4 and 25 m depth, and attained its highest density at station 34 (90 ind.m^-2^).

#### Feeding.

Gut content analysis of digestive tracks of some worms revealed that this species mainly feeds on algae.

#### Reproduction.

One specimen has eggs in its coelomic cavity, measuring 200 µm in diameter on average. This species is known to be a simultaneous hermaphrodite, brooding large eggs (about 600 µm) in the parental tube (Pires et al. 2012; Arias et al. 2013).

#### Symbiotic relationship.

Specimens of *Diopatra marocensis* collected from stations D14 and 34 were densely infected by a parasite (?Entoprocta). The parasite is attached to the dorsal side, parapodia and branchiae of the worms. One specimen (ESFM−POL/2009−34) with 15 chaetigerous segments (posterior part is missing) had almost 40 parasites. Arias et al. (2010) found a symbiotic relationship between specimens of *Diopatra marocensis* and a peritricous protozoan (the genus *Epistylis*) from northern Spain.

#### Geographical distribution.

This species is only known from the East Atlantic (near Gibraltar; Morocco, Spain and Portugal) and the eastern Mediterranean. The presence of this species in the eastern Mediterranean and absence in the western Mediterranean is interesting. There could be two reasonable explanations for its presence in the eastern Mediterranean; 1) this species might have been introduced to the area by ballast water of ships; 2) it might be an Atlanto-Mediterranean species that widely occurs near openings of river mouths in the area, but has been overlooked up to now, or misidentified as a juvenile specimen of the large Mediterranean species *Diopatra neapolitana*. These explanations can be refuted or proved when more data regarding this species in different basins of the Mediterranean are accumulated. As there is a big gap between the Atlantic and Mediterranean populations of this species, this species can be regarded as a new alien species for the Mediterranean Sea, at least for now.

#### Discussion.

*Diopatra marocensis* can be distinguished from other *Diopatra* species (*Diopatra neapolitana*, *Diopatra marocensis*, *Diopatra micrura*, *Diopatra biscayensis* and *Diopatra cryptornata*) reported from the north-eastern coast of the Atlantic Ocean and Mediterranean Sea, in having pectinate chaetae that have oblique combs with 16−18 thin teeth. The original description of *Diopatra marocensis* differs from the re-description of the species based on the eastern Mediterranean specimens in having different pigmentation in the anterior end (generally pale, but small specimens having palps with irregular brownish specks, and peristomium and anterior chaetigers with mid-dorsal bars on the posterior part of segment) and branchiae with fewer numbers of whorls (max. 8−9 whorls in the original description vs. 15 in the eastern Mediterranean specimens). However, such characters of the eastern Mediterranean specimens were also noted on the Spanish specimens (H. Paxton, pers. comm.).

## Supplementary Material

XML Treatment for Diopatra marocensis
